# Myelin Plasticity and Repair: Neuro-Glial Choir Sets the Tuning

**DOI:** 10.3389/fncel.2020.00042

**Published:** 2020-02-28

**Authors:** Remi Ronzano, Melina Thetiot, Catherine Lubetzki, Anne Desmazieres

**Affiliations:** ^1^Institut du Cerveau et de la Moelle épinière, Sorbonne Universités UPMC Université Paris 06, CNRS UMR7225-Inserm U1127, Paris, France; ^2^Unit Zebrafish Neurogenetics, Department of Developmental & Stem Cell Biology, Institut Pasteur, CNRS, Paris, France; ^3^Assistance Publique-Hôpitaux de Paris, Hôpital Pitié-Salpêtrière, Paris, France

**Keywords:** myelin, oligodendrocytes, glia, microglia, astrocyte, myelination, plasticity, remyelination

## Abstract

The plasticity of the central nervous system (CNS) in response to neuronal activity has been suggested as early as 1894 by Cajal (1894). CNS plasticity has first been studied with a focus on neuronal structures. However, in the last decade, myelin plasticity has been unraveled as an adaptive mechanism of importance, in addition to the previously described processes of myelin repair. Indeed, it is now clear that myelin remodeling occurs along with life and adapts to the activity of neuronal networks. Until now, it has been considered as a two-part dialog between the neuron and the oligodendroglial lineage. However, other glial cell types might be at play in myelin plasticity. In the present review, we first summarize the key structural parameters for myelination, we then describe how neuronal activity modulates myelination and finally discuss how other glial cells could participate in myelinic adaptivity.

## Introduction

Myelin is a feature of jawed vertebrates (Zalc et al., 2008), though it has also been acquired independently along with evolution by few invertebrate taxa (Hartline and Colman, 2007). Myelin is formed by lipid-rich membrane layers wrapped around axons, providing electrical insulation and metabolic support. This process ensures fast saltatory conduction (Waxman and Foster, 1980), reaching velocities that would otherwise require giant axons (Hartline and Colman, 2007). Despite its energy cost (Harris and Attwell, 2012), myelin correlates with increased population fitness, more efficient behaviors and increased body size.

*In vitro* and *in vivo* models showed that the axonal diameter is a key determinant for myelination (Lee S. et al., 2012; Goebbels et al., 2017; Mayoral et al., 2018). The usual threshold for myelinated axon in the peripheral nervous system (PNS) is 1 micron (Matthews, 1968). However, theoretical predictions suggest that myelination can increase axonal conduction with a diameter as low as 0.2 μm (Waxman and Bennett, 1972), which fits with central nervous system (CNS) myelination, where axons with diameters from 0.4 μm can be myelinated (Hildebrand et al., 1993). At a given axonal diameter, the conduction velocity of an action potential depends on the structural characteristics of myelin. The major parameters are the g-ratio (the axonal diameter divided by the total outer diameter of the fiber; Smith and Koles, 1970), and the internodal length (Huxley and Stampfli, 1948). Mean measured value and predicted optimum for the g-ratio are between 0.6 and 0.7 in the PNS and slightly above in the CNS white matter (Rushton, 1951; Smith and Koles, 1970; Waxman and Swadlow, 1976; Michailov et al., 2004; Chomiak and Hu, 2009). The conduction velocity also increases with the internodal length until it reaches a plateau at 1,000 μm (Brill et al., 1977; Moore et al., 1978). In the PNS, the majority of internodes exceed 500 μm (Hildebrand et al., 1994), and variations in internodal length have little effect on conduction velocity (Wu et al., 2012; Simpson et al., 2013). In the CNS, internodes are much shorter, on average 50 μm in gray matter and 150 μm in white matter (Tomassy et al., 2014; Arancibia-Cárcamo et al., 2017; Stedehouder et al., 2017, 2019), and changes in their length have a higher impact on conduction velocity (Etxeberria et al., 2016). Thus, in the CNS, structural characteristics allow for modulation of conduction velocity.

In the CNS, *in vitro* (Watkins et al., 2008) as well as *in vivo* experiments (Czopka et al., 2013) have demonstrated that myelinating oligodendrocytes (OLs) establish myelin sheaths in only a few hours. Following this step, between 20 and 60 myelin sheaths per OL are stabilized in rodents (Matthews and Duncan, 1971; Chong et al., 2012), and about 15 per OL in zebrafish. The deposition of the successive myelin layers is led by the inner tongue which wraps around the axon and extends laterally (Snaidero et al., 2014). The dynamics of the actin cytoskeleton appears finely regulated to trigger myelin wrapping, with an actin polymerization at the leading edge of the inner tongue and subsequent depolymerization (Nawaz et al., 2015; Zuchero et al., 2015). Moreover, defects in adhesion molecules expressed at myelin membranes and axolemma affect the number, the length and the folding of myelin sheaths, disrupting target recognition and myelin extension around and along axons (Djannatian et al., 2019; Hughes and Appel, 2019; Klingseisen et al., 2019).

Myelination has long been viewed as a process ending in young adults. However, in the CNS, though some structures like the optic nerve are fully myelinated (Honjin et al., 1977; Bartsch et al., 1997; Dangata and Kaufman, 1997), most of the areas exhibit partial myelination. The corpus callosum contains 20–40% of unmyelinated fibers in adult rodents (Seggie and Berry, 1972; Gravel et al., 1990; Olivares et al., 2001), and the myelination profile of excitatory as well as inhibitory neurons show discontinuous patterns in the cortical and hippocampal areas (Tomassy et al., 2014; Micheva et al., 2016; Stedehouder et al., 2017, 2019). These myelination patterns have been suggested to regulate action potentials (APs) arrival at the presynaptic compartment (Salami et al., 2003) and provide metabolic support to fast-spiking neurons that have a high energy demand (Micheva et al., 2016). Incomplete myelination should allow for myelin plasticity, which could potentiate specific connections or provide additional metabolic support in the CNS by the addition of myelin on specifically activated networks.

## Neuronal Activity Shapes Myelination Profile Along With Life

The role of neuronal activity in modulating myelination was first suggested more than 50 years ago by the effect of light deprivation on mouse optic nerves (Gyllensten and Malmfors, 1963). Later on, modulation of the oligodendroglial lineage through neuronal activity was shown *in vitro* using neurotoxins and electrical stimulations (Barres and Raff, 1993; Demerens et al., 1996; Fields and Stevens, 2000; Stevens et al., 2002). More recently, the relationship between these processes has been extensively studied with growing evidence that neuronal activity plays a key role in the modulation of every step of myelination both during development and in adulthood.

### The Oligodendroglial Lineage Can Perceive the Neuronal Activity

**Figure 1:**
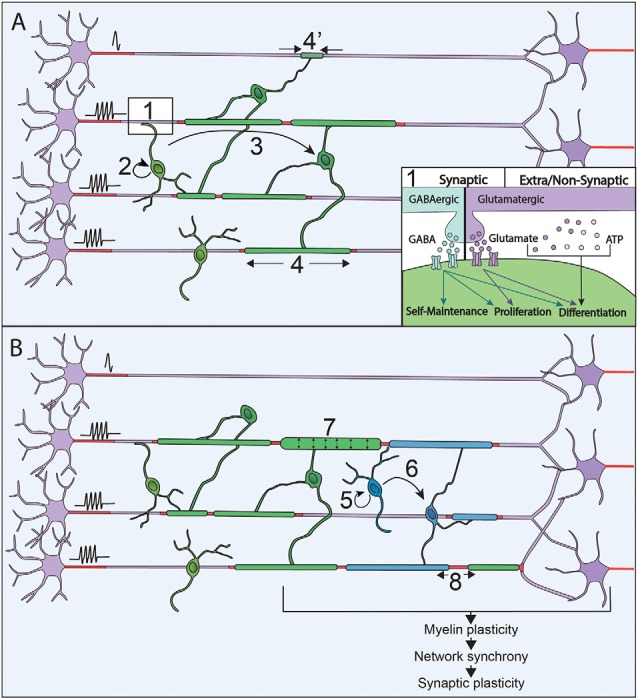
Neuronal activity modulates myelination processes along with life. **(A)** Neuronal activity is sensed through synapses and extra/non-synaptic junctions between neurons and oligodendrocyte progenitor cells (OPCs; 1). Neurons release GABA (blue) or Glutamate (purple) that activate respectively GABA_A_ and AMPA receptors at neuron-OPC synapses. Vesicles of ATP (orange) can also be released by neurons and modulate OPCs physiology at extra-synaptic and non-synaptic junction together with glutamate. Neuronal activity modulates every step of myelination during development: OPCs maintenance and proliferation (2), OPCs differentiation in OLs (3) and myelin sheaths stabilization and extension (4 and 4’). **(B)** In the adult, OPCs are maintained and their proliferation (5), as well as differentiation (6), can be promoted by an increase of neural activity when performing a new task. This increase in neuronal activity can also modulate the characteristics of myelin sheaths that are already formed by increasing their thickness (7) and modifying the nodal gaps (8).

Neuronal activity can modulate oligodendrocyte progenitor cells (OPCs) proliferation, maintenance and differentiation in zebrafish and mammals (Hill et al., 2014; Zonouzi et al., 2015; Hamilton et al., 2017; Hoche et al., 2019). Glutamatergic and GABAergic neurons have been shown to form bona fide synapses on OPCs in rodents (Bergles et al., 2000; Lin and Bergles, 2004) and humans (Gallo et al., 2008), with neuronal inputs on OPCs being consistent between brain regions (Mount et al., 2019). The activity of afferent neurons through the activation of either AMPA or GABA receptors is widely involved in the control of OPCs fate and self-maintenance along CNS development (Mangin et al., 2012; Zonouzi et al., 2015; Balia et al., 2017; Kougioumtzidou et al., 2017; Chen et al., 2018; Figure 1). Furthermore, OPCs are not only sensitive to the presence of neuronal activity, but also to the pattern of activity, which modulates differently their proliferation and differentiation (Nagy et al., 2017). Although the involvement of neuron-OPCs synapses has been largely documented, non-synaptic junctions between neurons and OPCs have also been involved in the facilitation of OPCs differentiation *in vitro* (Wake et al., 2015; Figure 1). The control of OPCs proliferation and differentiation has been showed to depend on Ca^2+^ signals triggered by neuronal activity *in vitro* in rodents (Wake et al., 2011) and *in vivo* in zebrafish (Hoche et al., 2019). However, depending on the developmental stage and the anatomical area studied, OPCs respond differently to neuronal activity, possibly related to their heterogeneous expression of voltage-gated channels and receptors to neurotransmitters (Káradóttir et al., 2008; Hoche et al., 2019; Spitzer et al., 2019).

### Neuronal Activity Modulates Axon Selection as Well as Myelination Pattern

Highly specific selection of the axonal segments to be myelinated is necessary to lead to adequate myelination patterns. It has been shown *in vitro* and *in vivo* in mice and zebrafish that the choice of the target axons is promoted by neuronal activity (Hines et al., 2015; Wake et al., 2015; Mitew et al., 2018; Figure 1). In zebrafish, the maintenance of nascent myelin sheaths is increased on electrically active axons (Hines et al., 2015). Neuronal activity can also regulate the number of myelin sheaths per OL in zebrafish (Mensch et al., 2015) and their length in mouse optic nerves (Etxeberria et al., 2016). Activity-dependent myelination acts through the release of axonal vesicles (Hines et al., 2015; Mensch et al., 2015; Wake et al., 2015; Etxeberria et al., 2016) triggering Ca^2+^ signals in OLs. In zebrafish, Ca^2+^ signals along myelin sheaths regulate their stabilization and growth in an axonal activity-dependent manner (Baraban et al., 2018; Krasnow et al., 2018). The frequency, the duration and the amplitude of Ca^2+^ signals appears to be crucial for myelination and correlates with axonal activity (Krasnow et al., 2018). Based on what has been done on NG2 cells (Nagy et al., 2017), deciphering the effects of various neuronal firing patterns on OLs myelination may result in a better understanding of these complex modulations. However, the prominence of neuronal activity in the control of myelination needs to be weighted, as myelin increase could also reflect concurrent growth of axonal arborization (Stedehouder et al., 2018). Moreover, non-neuronal activity related mechanisms concomitantly participate to axon selection during myelination (Rosenberg et al., 2008; Bechler et al., 2018; Mayoral et al., 2018) and, for some neuronal populations, myelination occurs independently of neuronal activity (Koudelka et al., 2016). It can, therefore, be considered that neuronal activity is rather acting as a modulator allowing to adapt myelination pattern to the activity of the neuronal networks.

### Myelination in Adulthood as an Adaptive Mechanism

In mice, OPCs keep proliferating and differentiating in adult CNS, with 5–20% of OLs generated during adulthood (Rivers et al., 2008; Kang et al., 2010; Simon et al., 2011; Young et al., 2013). The OLs generated in adulthood could contribute to cellular turnover or adaptive myelination. However, in mice, except in the optic nerves, OLs survival rate is over 90% at 8 months suggesting that the new OLs generated may rather participate in adaptive processes (Tripathi et al., 2017). Remodeling of existing myelin has first been observed, in social isolation of adult mice, where induction of behavioral changes correlate with myelin sheath thinning and transcriptional changes in OLs in the medial prefrontal cortex (Liu et al., 2012). Myelin plasticity could further be associated with changes in internodal or nodal gap length, both of which have been described to tune conduction velocity (Ford et al., 2015; Arancibia-Cárcamo et al., 2017; Figure 1). Indeed, myelin sheath length can be remodeled once it is established; however, these changes are relatively rare in adulthood and sensory enrichment failed to induce any measurable changes in sheath length in rodents (Hill et al., 2018; Hughes et al., 2018). Alternatively, conduction velocity could be tuned by changes in nodal gap length, which can be modulated in adult mice (Dutta et al., 2018), upon neuronal activity changes (Cullen et al., 2019; Korrell et al., 2019).

So far, adaptive myelination has mainly been associated with the generation of new OLs and the addition of new myelin sheaths (Figure 1). First, the learning of complex motor tasks has been shown to trigger OPCs proliferation, OLs maturation and myelin deposition (Sampaio-Baptista et al., 2013; McKenzie et al., 2014). Furthermore, in the same paradigm of complex wheel running, OPCs differentiation occurred within the range of a few hours (Xiao et al., 2016). Relatively short optogenetic stimulations of the premotor areas at a physio mimetic frequency triggered OPCs proliferation, oligodendrogenesis and myelin thickening, coupled to behavioral improvement (Gibson et al., 2014), corroborating the involvement of adaptive myelination in motor learning. Lastly, spatial learning was shown to trigger adaptive myelination, and impairment in adaptive myelination leads to defect in memory consolidation (Steadman et al., 2020) and short term memory (Geraghty et al., 2019). In humans, a link between neuronal activity and the addition of new myelin sheaths in adult CNS has been shown by studies on healthy subjects achieving motor and memorization tasks. White matter microstructural changes were demonstrated (Scholz et al., 2009; Takeuchi et al., 2010), and the amplitude of the effect correlated with the training duration (Taubert et al., 2010). These changes could be due to myelin deposition *per se* or reflect axonal remodeling (Zatorre et al., 2012). The origin of the newly added myelin has been investigated by immunohistochemical studies, which provided evidence of proliferating OPCs in the adult brain (Geha et al., 2010). This was further supported by studies on non-human primates showing an increase in the number of OLs during adulthood (Peters and Sethares, 2004; Peters et al., 2008). Alternatively, myelin could also arise from pre-existing OLs persisting into adulthood, as identified in humans (Yeung et al., 2014; Fard et al., 2017; Jäkel et al., 2019). Thus, although adaptive myelination also occurs in the human brain, to which extent mechanisms are shared between rodents and humans is still under debate.

Myelin adaptation could be involved in the fine-tuning of neural network synchrony, and action potential arrival at the presynaptic compartment (Pajevic et al., 2014; Ford et al., 2015), that are thought to govern learning and memory (Feldman, 2012; Kandel et al., 2014; Korte and Schmitz, 2016). The effect of adaptive myelination on short term memory and memory consolidation supports this hypothesis (Geraghty et al., 2019; Steadman et al., 2020), but future studies will be needed to determine how adaptive myelination modulates the electrophysiological parameters of specific parts of neuronal circuits, and further creates a synchronization at specific connections. Moreover, feedback signals from the myelinated axon/neuron allowing for the fine control of myelin addition and removal should be required to tune finely AP arrival at the synapses and further synchronize the circuits. Until now, they remain unknown, with previous works on synaptic plasticity being a potential source of inspiration to investigate them (Fields et al., 2014).

Newly added myelin sheaths could further provide metabolic support to axons (Fünfschilling et al., 2012; Lee Y. et al., 2012; Meyer et al., 2018), the metabolic supply being regulated by neuronal activity (Saab et al., 2016). This myelin addition probably would not result in a global energetic advantage (Harris and Attwell, 2012), but might be needed to generate fast-spiking firing discharges and thus allow for precise axonal firing (Micheva et al., 2016; Moore et al., 2019).

Although the molecular mechanisms inducing adaptive myelination in the adult are still unclear, recent studies showed the involvement of two factors, endothelin (Swire et al., 2019) and BDNF (Geraghty et al., 2019). Neuronal activity triggers an increase in blood flow that in turn increases endothelin expression by endothelial cells (Walshe et al., 2005; Pandit et al., 2015). This has been shown to increase myelination *ex vivo* (Yuen et al., 2013). In adult mice, endothelin rescues myelination defects triggered by social isolation, thus confirming its involvement in adaptive myelination (Swire et al., 2019). BDNF had first been suggested to modulate activity-dependent myelination (Lundgaard et al., 2013) and later showed to be a regulator of adaptive myelination (Geraghty et al., 2019). It is produced by neurons in an activity-dependent manner (Balkowiec and Katz, 2000; Hartmann et al., 2001; Dieni et al., 2012) and can be released by synaptic vesicles (Park et al., 2014). Thus, BDNF secretion could specifically trigger adaptive myelination along activated axons. However, BDNF is not only released by neurons, but also by astrocytes (Fulmer et al., 2014; Zhang et al., 2014) and microglial cells (Parkhurst et al., 2013). These complex BDNF signals might have to be integrated by the oligodendroglial lineage when it comes to adaptive myelination, as well as in injury (McTigue et al., 1998; Ikeda et al., 2002; Ramos-Cejudo et al., 2015). Lastly, OPCs themselves could modulate myelination and myelin plasticity directly or indirectly, in particular through the secretion of BDNF or retinoic acid (Tanaka et al., 2009; Parolisi and Boda, 2018; Goncalves et al., 2019). Adaptive myelination and repair should thus not be considered only as direct neuronal crosstalk with the oligodendroglial lineage, but also in regard to their direct cellular environment.

## Myelination and Repair Are Also Modulated by Other Neuro-Glial Interactions

The crosstalk between neuron and glia is complex and probably critical when it comes to myelination regulation, in adaptive processes and repair. Astrocytes and microglial cells are known to participate in (re)myelination modulation and have been described to detect neuronal activity (for review, Domingues et al., 2016; Adaikkan and Tsai, 2019; Bar and Barak, 2019; Molina-Gonzalez and Miron, 2019). Although astrocytes and microglia may be involved in molecular mechanisms modulating adaptive myelination, the understanding of their impact on adult myelination processes is still limited.

### Control of Myelination and Myelin Plasticity by Astrocytes

Astrocytes are the most abundant CNS glial cell type, with a major role in metabolic support, homeostatic functions, assembly and modulation of synapses, Blood-Brain Barrier (BBB) integrity and nervous tissue scaring. They further participate in neuronal activity and myelination regulation, in plasticity and learning (for review, Barres, 2008; Fields et al., 2014). Astrocytes are heterogeneous, with protoplasmic astrocytes, in the gray matter, interacting with synapses and BBB, and fibrous astrocytes, in the white matter, contacting nodes of Ranvier and blood vessels (for review, Sofroniew and Vinters, 2010).

**Figure 2:**
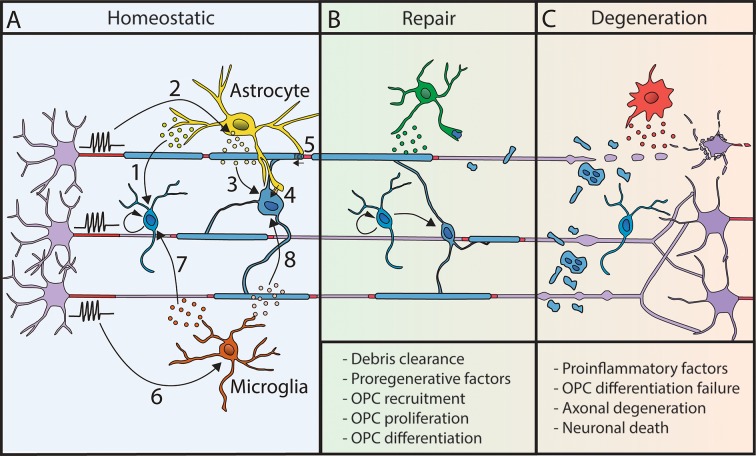
Myelination processes are modulated by other glial cells. **(A)** In homeostatic conditions, astrocytes and microglial cells modulate myelin deposition. Astrocytes release factors regulating OPC proliferation (1). Neuronal activity triggers LIF release by astrocytes (2), which promotes myelination (3). Moreover, astrocytes are metabolically coupled to OLs (4) and modulate conduction velocity by acting on myelin thickness and nodal length (5). Microglial cell behavior is modulated by neuronal activity (6). They release factors that promote OPC proliferation and differentiation (7) and activate myelination (8). **(B)** Following demyelination, glial cells can promote repair by the clearance of myelin debris and the release of pro-regenerative factors. **(C)** However, their sustained proinflammatory activity can lead to repair failure and neurodegeneration.

Astrocytes have been described to regulate all oligodendroglial lineage steps, from OPCs proliferation to differentiation and myelination (for review, Domingues et al., 2016; Figure 2), in particular by secretion of various factors such as IGF1, CNTF, CXCL1, TIMP-1 and LIF (Gard et al., 1995; Stankoff et al., 2002; Ye et al., 2004; Padovani-Claudio et al., 2006; Modi et al., 2013; Jiang et al., 2016). Astrocytic role in myelination is partly dependent on neuronal activity, with the activity-dependent neuronal release of ATP triggering the secretion of astrocytic LIF factor, which further promotes OL survival and myelination (Ishibashi et al., 2006). Astrocytes also provide some lipids necessary to support the metabolic costs of myelination (Camargo et al., 2017) and promote OLs survival and maturation through direct physical contacts (Sakurai et al., 1998; Corley et al., 2001). They further connect with oligodendrocytes through connexins necessary for myelin maintenance and support of OLs K^+^ buffering during neuronal activity (Menichella et al., 2006; Orthmann-Murphy et al., 2008; Tress et al., 2012). Once myelin is formed, astrocytes further play a role in myelin plasticity by regulating myelin thickness and nodal gap length (Dutta et al., 2018). Lastly, astrocytes control local blood flow depending on neuronal activity (for review, Nortley and Attwell, 2017) and could thus further be involved in the indirect control of adaptive myelination by vasculature (Swire et al., 2019).

Astrocytes also play a complex role in demyelination and repair (Figure 2). They have been described to be rather beneficial *in vitro*, as well as *in vivo*, in chemically-induced demyelinating mouse models (Franklin et al., 1991; Selvaraju et al., 2004; Kramann et al., 2019). Following demyelination, they attract OPCs, promote their proliferation and differentiation (Omari et al., 2005; Patel et al., 2012). In contrast, astrocytes might play an inhibitory role in remyelination, in particular by inhibiting OLs maturation (Blakemore et al., 2003; Back et al., 2005; Sloane et al., 2010). They can further promote proinflammatory responses, circulating immune cell recruitment through BBB and modulate the number of activated microglial cells (Brambilla et al., 2014; Kim et al., 2014; Eilam et al., 2018). The complex role played by astrocytes, related to their phenotype, further depends on environmental cues and interaction with surrounding cells (Liddelow et al., 2017).

### Control of Myelination and Myelin Plasticity by Microglia

Microglial cells are the resident immune cells of the CNS, where they represent 5–10% of the cells (Lawson et al., 1990). They continually monitor their environment (Nimmerjahn et al., 2005), and play complex roles in neuroplasticity, homeostasis, host defense, healing, debris clearance and peripheral cell recruitment (for review, Colonna and Butovsky, 2017; Prinz et al., 2019). They can adopt different phenotypes, with environment-dependent transcriptional profiles (Gosselin et al., 2014, 2017), and proinflammatory to pro-regenerative polarization (Miron and Franklin, 2014), though a strict dichotomy is an inadequate vision (Ransohoff, 2016). Microglial cells are further sensitive to neuronal activity (Li et al., 2012; Liu et al., 2019; Stowell et al., 2019; Cserép et al., 2020). Altered microglia activity at different stages of life is associated with developmental and acquired neurological pathologies and can impair the plasticity-related process and cognitive function (Morris et al., 2013).

In homeostatic condition, microglia can support survival, differentiation, myelinogenesis, and homeostasis of the oligodendroglial lineage (Hamilton and Rome, 1994; Butovsky et al., 2006; Pasquini et al., 2011; Shigemoto-Mogami et al., 2014; Hagemeyer et al., 2017; Wlodarczyk et al., 2017; Figure 2). Activated microglia associated with myelin deficits has further been described in neurodevelopmental disorders and mental conditions (Garey, 2010; Morgan et al., 2010; Janova et al., 2018; Bar and Barak, 2019; Barak et al., 2019). These defects might be partly related to a lack of adaptive myelination. Indeed, microglia activation state is modulated by neuronal activity (Iaccarino et al., 2016; Adaikkan et al., 2019; Giorgetti et al., 2019; Martorell et al., 2019; Garza et al., 2020), and has been shown to modulate adaptive myelination in adult (Geraghty et al., 2019).

In demyelinating diseases, microglial activation is an early hallmark in multiple sclerosis (MS) together with axonal damage even prior to demyelination (Howell et al., 2010; Nikić et al., 2011). Microglia can have a dual role in repair, either impairing or promoting myelination in MS and its models in rodents (for review, Miron, 2017) depending on its phenotype (proinflammatory or pro-regenerative; Miron et al., 2013; Locatelli et al., 2018). It is considered that the pro-regenerative/pro-remyelinating effect of microglia might be related both to the secretion of pro-myelinating factors and the capacity of myelin debris clearance (Lampron et al., 2015; Cantuti-Castelvetri et al., 2018; Figure 2). Astrocytes can further participate in microglial recruitment at the lesion to promote debris clearance (Skripuletz et al., 2013), taking part in a global crosstalk. Reciprocally, the effect of extracellular vesicles produced by microglia on OPCs is modulated by astroglia (Lombardi et al., 2019). Finally, it has been recently described that microglial activation following cancer therapy can lead to astroglial activation and alter adaptive myelination highlighting the importance of inter-glial communication in these mechanisms (Gibson and Monje, 2019; Gibson et al., 2019).

The complex contribution of activated astrocytes and microglia in inflammatory conditions thus makes them key players in repair, able to either compromise or promote the efficacy of myelin redeposition (Franklin and Goldman, 2015). The activation states of these cells were further modulated by neuronal activity, the characterization of the complex crosstalk between glial and neuronal partners should pave the way to a better understanding of myelinic regulation and to more integrative therapeutical strategies.

## Author Contributions

RR, AD, and CL wrote the manuscript and made the figures. AD, RR, CL, and MT proofread the manuscript.

## Conflict of Interest

The authors declare that the research was conducted in the absence of any commercial or financial relationships that could be construed as a potential conflict of interest.
